# A conversation with David Maahs, MD, PhD, Lucile Salter Packard Professor of Pediatrics, Stanford University

**DOI:** 10.1017/cts.2025.10165

**Published:** 2025-10-03

**Affiliations:** Clinical Research Forum, Washington, DC, USA

## Top 10 Clinical Research Achievement Awards Q & A

This article is part of a series of interviews with recipients of Clinical Research Forum’s 2025 Top 10 Clinical Research Achievement Awards. This interview is with David Maahs, MD, PhD, Lucile Salter Packard Professor of Pediatrics, Stanford University. Dr Maahs’s research focuses on improving care and preventing complications in people with type 1 diabetes (T1D). He has conducted studies to develop artificial pancreas systems, improve glucose control, lower disease burden, prevent the complications of diabetes, and reduce disparities in diabetes care. He received a 2025 Top 10 Clinical Research Achievement Award for “Teamwork, Targets, Technology and Tight Control (4T) Study.” *The interview has been edited for length and clarity.*

## When did you first get interested in clinical research?

My first job out of residency was being a pediatric hospitalist. That job taught me a lot about clinical care, and I also learned that if I wanted to improve the care we provided, I would need to do research. Fortunately, I had the opportunity to work with the adult endocrinology chief, Dr David Schade, on a randomized controlled trial, and that mentored project, along with being a hospitalist, got me really excited about doing clinical research. After that, I went on to complete a pediatric endocrinology fellowship and a PhD in epidemiology.

## How do you combine your training in medicine, epidemiology, and research?

I try to corroborate what I see in clinic with large epidemiology datasets and registry datasets on patient outcomes and then use that to develop hypotheses that can be tested to try to come up with improved care. Over the past 10–15 years, I’ve been fortunate to work on diabetes technology, so I’ve been able to see and be a part of such dramatic improvements, going from finger pokes and shots multiple times a day to now having continuous glucose monitors, automated insulin delivery, and other technologies that enable people with diabetes to have a much better quality of life.

## That brings us to the award-winning research. What did you investigate in the “Teamwork, Targets, Technology and Tight Control (4T) Study?”

We prospectively assessed the impact of a systematic and equitable digital-health, team-based care program implementing tighter glucose targets, early use of continuous glucose monitoring (CGM), and remote patient monitoring on glycemia in young people with newly diagnosed T1D.

## What did the results show?

Clinical implementation of the study helped participants meet glucose targets and improved glycemia without unexpected serious adverse events. That means the strategies in the 4T Study can be used for better and more equitable care for individuals with T1D. For example, it’s standard of care now to start CGM early, but with that you get 288 glucose readings in a day—and that’s a lot of data. Studies like this demonstrate how to use that data in a way that helps people get the care they need, so we don’t have to rely on the historic model of “call us if you have a problem and we’ll see you in three months.”

## The title of the study highlights “teamwork, targets, tech, and tight control.” Which of these is most important for effective diabetes management?

The first “t” of “teamwork” is by far the most important—and it encompasses teamwork between the child and the parent, between the child, the parent, and the care team, and also between all the members of the care team. Care teams are now quite extensive and can include diabetes educators, who can be dieticians or nurses, diabetes psychologists, exercise physiologists, dieticians, and more. These are all key roles, and everyone needs to work together to achieve positive outcomes.

## In the 4T Study, how did members of the care team collaborate?

One of the most innovative aspects of our research is the dashboard that we developed with our engineering team led by Dr David Scheinker. We call it “TIDE,” short for “timely interventions for diabetes excellence,” and it’s an open-source algorithm-enabled care model that provides patient prioritization. TIDE saves time for diabetes educators and enables them to prioritize who needs attention between visits, so patients can get the care they need when they need it. That way, small problems can be dealt with early, before they become bigger problems. It also allows diabetes educators to work at the top of their certification, providing education, as well as dose adjustments.

## Where is this research heading next?

The next iteration of the study—the “4T Study 2”—just finished a few weeks ago, and it tests if outcomes are impacted by reducing the amount of patient contacts. We are also interested in seeing the impact of putting more emphasis on starting automated insulin delivery earlier. In addition, we had a workshop at Stanford in January with 15 pediatric diabetes centers who are part of the Type 1 Diabetes Exchange Quality Improvement Collaborative, and the focus was to explain our research and talk about adapting what we did to other sites. We want to see if we can get similar benefits after scaling up the program to 15 sites instead of just one.

## What are the challenges associated with implementing this program at other sites?

There’s a startup cost and that’s always a challenge; however, we researched costs and published a paper about the very positive business case for this program. There are also challenges with data integration so that all the different technologies like CGMs and insulin pumps can communicate with the TIDE dashboard. We met that challenge by working with the diabetes technology non-profit Tidepool and developing a turnkey solution that’s now available to any clinic in the country. Lastly, I think the biggest obstacle to implementing the program at other sites is just change management. As humans, we’re resistant to change, but the Type 1 Diabetes Exchange Quality Improvement Collaborative was formed to improve the quality of diabetes care, and people in that group want to do better and learn from each other. With their help we want to facilitate even more positive outcomes and make improvements faster.

## What advice do you have for people who are beginning their careers in clinical research?

It’s critical that you have encouragement and support when you’re starting out, and that’s why I’ve been so involved with NIH’s multi-center “Diabetes-Docs: Physician-Scientist DiabDocs-K12 Program” (DiabDocs), which is dedicated to the career development of physician-scientists who want to focus on basic and clinical diabetes. DiabDocs is now in its fourth year, and it’s open to scholars who can conduct research at any eligible U.S. institution, essentially enabling them to have a mentored research training experience at their local institution, but also be part of a national network. Having good mentoring and the protected time to be able to develop your research skills is essential. Plus, another thing that’s been so beneficial with DiabDocs is the peer support that participants have been able to give each other. There’s just so much value to getting to know other researchers who can give you advice and support.

## What’s the best part of your job as a clinical researcher?

It’s incredibly gratifying to see the benefits that research can provide for patients and their families. Over the past 20 years, I’ve been able to see how much better children, adolescents, and young adults with T1D do because they’ve got continuous glucose monitors and automated insulin delivery. The safety of the interventions has improved, glucose outcomes, and quality of life have improved, and we know that the long-term complications are going to be greatly reduced. All this has happened because JDRF (Juvenile Diabetes Research Foundation, now Breakthrough T1D) and NIDDK (National Institute of Diabetes and Digestive and Kidney Diseases) invested so heavily in studies back in the early 2000s. There’s a direct line from those research investments to where we are now, and it’s really rewarding to be part of how much has changed for children with T1D.

